# A patient-reported questionnaire for evaluating utilization of the National Essential Public Health Service Program in China among older adults with hypertension and diabetes

**DOI:** 10.3389/fpubh.2025.1459754

**Published:** 2025-03-11

**Authors:** Lu Liu, Meicen Liu, Linghe Yang, Xinyue Chen, Yuanli Liu, Lili You

**Affiliations:** ^1^School of Health Policy and Management, Chinese Academy of Medical Sciences and Peking Union Medical College, Beijing, China; ^2^National Cancer Center, Chinese Academy of Medical Sciences, Beijing, China

**Keywords:** health management, older adults, hypertension, diabetes, construct validity, confirmatory factor analysis

## Abstract

**Objectives:**

This study aimed to develop a patient-reported questionnaire to assess service utilization and patient satisfaction among older adults with hypertension and diabetes in primary health-care centers operating under China’s National Essential Public Health Service Program (NEPHSP).

**Methods:**

The questionnaire’s item pool was constructed on the basis of a logic model. A cross-sectional survey was conducted in three provinces of mainland China between November and December 2019. The questionnaire was evaluated using Cronbach’s alpha coefficients and confirmatory factor analysis (CFA) to refine items and assess internal consistency, construct validity, convergent validity, and discriminant validity.

**Results:**

The final questionnaire adopted a second-order factor model comprising three domains: essential services for all older adults, follow-up services for older adults with hypertension or diabetes, and patient satisfaction assessment. Through a two-step refinement process, nine factors encompassing 29 items were selected, including elements such as “health records and health education,” “blood pressure/glucose monitoring,” and “health education in follow-up.” Cronbach’s alpha coefficients indicated excellent reliability, with values of 0.899 and 0.906. The 29-item instrument had robust model fit for both hypertension and diabetes cohorts. The fit indices for the hypertension model included a Bollen–Stine bootstrap chi-square to degrees of freedom ratio (χ^2^/df) of 1.78, root mean square error of approximation (RMSEA) of 0.03, and goodness-of-fit index (GFI) of 0.97. Similarly, for the diabetes model, the fit indices were a Bollen–Stine bootstrap χ^2^/df ratio of 1.38, RMSEA of 0.02, and GFI of 0.97. CFA revealed factor loadings ranging from 0.516 to 0.940 for the hypertension model and from 0.504 to 0.943 for the diabetes model. All three first-order factors were significantly correlated with each other (*p* < 0.01), and their correlation coefficients were lower than the square root of the average variance extracted. The models demonstrated strong structural validity, convergent validity, and discriminant validity.

**Conclusion:**

A valid and reliable questionnaire for evaluating service utilization and patient satisfaction among older adults with hypertension and diabetes in primary health-care center was developed in China. This instrument will serve as a practical tool for patient-reported assessments within the NEPHSP framework at primary health-care centers.

## Introduction

The rapid aging of the population and shifts in lifestyle patterns have led to a substantial increase in the prevalence of chronic noncommunicable diseases among older adults. Among these, hypertension and diabetes are particularly prominent, ranking as leading causes of all-cause mortality and disability worldwide (1–6). In China, the estimated number of adults with hypertension reached 245 million in 2020, with more than 50% of individuals aged ≥65 years affected by the condition (7). Additionally, China has the largest population of patients with diabetes globally (8). As of 2017, the country was home to approximately 260.4 million older adults, approximately 30% of whom were reported to have diabetes (9). These chronic diseases often lead to severe comorbidities, characterized by high rates of disability and mortality, imposing substantial burdens on families and society (10, 11). Consequently, the effective control and management of hypertension and diabetes among older adults have become critical public health challenges in China (12, 13).

In 2009, China initiated ambitious health reforms, one of which was the implementation of the National Essential Public Health Service Program (NEPHSP). This initiative aligns with the Basic Health Service Package and essential service packages promoted by the World Health Organization (3), which have been adopted in many countries worldwide (14, 15). The NEPHSP offers free services through service packages accessible to all citizens via a network of over 800,000 primary health-care centers (PHCCs), thereby providing primary health care to China’s population of 1.4 billion. The program prioritizes key populations, including maternal and child health groups; older adults; and patients with hypertension, diabetes, and severe mental disorders (16, 17). Funded by the Chinese government, the NEPHSP aims to address essential public health needs. As of 2019, the NEPHSP delivers 12 types of service packages to residents through PHCCs, which are categorized into population-based public health services for all residents and individual health management services targeted at key populations (18).

Population-based public health services under the NEPHSP comprise five service packages: (1) resident health records management, (2) health education, (3) vaccination, (4) reporting of infectious diseases and public health emergencies, and (5) family planning education and sanitary inspections for all residents. Additionally, individual health management services include seven service packages: (1) maternal health management, (2) children’s health management, (3) health management for older adults, (4) health management for patients with chronic disease (hypertension and type 2 diabetes), (5) health management for patients with severe mental disorders, (6) health management for patients with tuberculosis, and (7) health management using traditional Chinese medicine (TCM). These services, delivered through PHCCs, ensure that all residents have access to essential public health services that are affordable, equitable, and of high quality. This approach aligns with the goal of achieving universal health coverage, regardless of geographic location or socioeconomic status (19).

Patients with a confirmed diagnosis of hypertension aged ≥35 years who consented to health management by general practitioners in community health centers (CHCs) are enrolled in an electronic health record system. These patients subsequently receive comprehensive interventions provided by multidisciplinary teams comprising general practitioners, nurses, and public health doctors based at their local CHC (20). The services offered include screening, lifestyle and health status assessments, physical examinations, ancillary examinations, health checkups, and personalized health guidance, among others (18). Between 2009 and 2019, the NEPHSP achieved remarkable progress, with the health management rate for older adults with increasing by 26.23%, reaching 67.41% (21). During the mass disruptions to health systems caused by the SARS-CoV-2 pandemic, the NEPHSP played a pivotal role in maintaining continuity of care for these patients. Community-based measures implemented by PHCCs included long-prescription policies, follow-up visits by general practitioners via phone or video conferencing, and home delivery of medications facilitated by community health workers (22, 23).

To date, evaluations of the effectiveness of the NEPHS have primarily relied on monitoring data reported by government sources (24, 25). In this study, we developed a patient-reported questionnaire specifically designed to assess the utilization of health management services for patients with hypertension and diabetes within the NEPHSP. Notably, our focus extended beyond service utilization to also include patient satisfaction. This study developed a reliable and valid patient-reported questionnaire to accurately measure the utilization of and satisfaction with health management services for older patients in the community. This questionnaire will serve as a robust and valid instrument for health management service surveys in the primary health-care sector.

## Methods

### Item pool formation

The primary objective in developing the assessment instrument was to comprehensively generate items and domains that accurately capture the quality of services received by patients and their satisfaction levels. To achieve this, a systematic review of relevant literature databases and policy documents was conducted. The review focused on the *Code of the NEPHSP* (*Third Edition*) (18), the *National Guidelines for the Prevention and Control of Diabetes in Primary Care* (26), and the *National Guidelines for the Prevention and Control of Hypertension in Primary Care* (27). Based on this review, an expert panel was convened to provide insights. Through multiple rounds of expert consultations, a set of survey instrument domains and an item pool of 40 items were generated (Supplementary Table 1).

### Logical analysis

A logic model was employed to analyze the management service processes for older adults with hypertension or diabetes. Widely employed in the public health sector and increasingly adopted by various organizations outside the public health sector (28, 29), logic models are instrumental in meticulously tracking program activities from inception to completion. Logic models involve “modeling or simulating” real-life scenarios in a manner that highlights the underlying logic governing them. These models elucidate the causal relationships between different components of a program, offering a systematic approach to understanding the pathway toward achieving desired outcomes. These models comprise causal chains, which elucidate why certain phenomena occur, or fail to occur, through a series of manageable activities.

**Figure 1:**
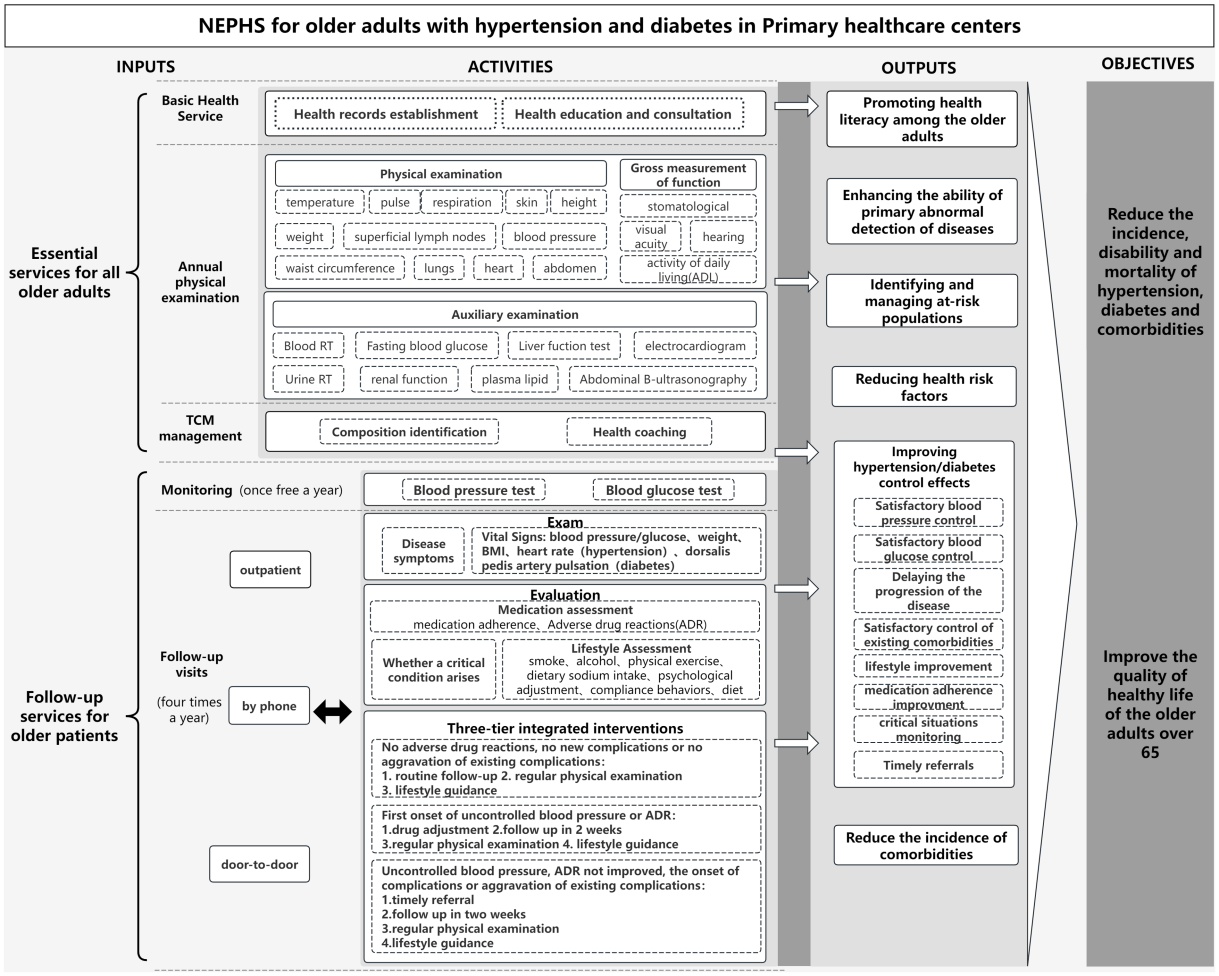
Logical model of management services for older adults with hypertension or diabetes under the NEPHSP.

Based on the *Code of the NEPHSP* (*Third Edition, 2017*) (18), we comprehensively summarized and analyzed the core components of service packages designed for older patients. These components includes inputs, such as service components; activities, which cover the implementation process and program outputs; and the program objectives (Figure 1). The services offered under the NEPHSP can be broadly categorized into two primary domains:

1. Essential services for all older adults: This domain encompasses the establishment of health records, health education, annual free physical examinations (including physical measurements, functional assessments, and auxiliary examinations), and TCM management. Physical examination includes measurements of temperature, pulse, respiration, blood pressure, height, weight, waist circumference, skin condition, the superficial lymph nodes, the lungs, the heart, the abdomen, gross oral cavity examinations, vision, hearing, and motor function. Auxiliary examinations include routine blood and urine tests, liver function tests (serum glutamic oxaloacetic transaminase, serum glutamic alanine transaminase, and total bilirubin), renal function tests (serum creatinine and blood urea), fasting glucose, lipid profile (total cholesterol, triglycerides, low-density lipoprotein cholesterol, and high-density lipoprotein cholesterol), electrocardiograms, and abdominal ultrasounds (hepatobiliary, pancreatic, and splenic assessments).
2. Follow-up services for older patients with diabetes or hypertension: These services encompass health monitoring, examinations, evaluations, and interventions provided through outpatient visits, home visits, or telephone consultations. Follow-ups are conducted four times a year.

These services actively promote health literacy and reduce health risk factors among older adults. This is achieved through the widespread implementation of electronic health records, resident health education lectures and consultations, identification of health components, and provision of TCM health coaching. Additionally, the program enhances disease detection capabilities at the grassroots level, enabling the early identification and management of high-risk populations through annual medical checkups, which include physical examinations, functional assessments, and auxiliary examinations. Furthermore, follow-up services contribute to more efficient management of hypertension or diabetes mellitus among older adults. During these follow-up services, health-care professionals at community or township health centers monitor patients’ vital signs and assess their disease symptoms, medication adherence, and lifestyles. Through a three-tiered integrated disease management approach, these health-care workers help improve blood pressure or glucose control, promote medication adherence and healthy lifestyle behaviors, delay disease progression, and ensure timely referrals for patients with severe or critical conditions. This integrated strategy helps reduce the incidence of complications associated with chronic diseases. The objectives of the service package are to reduce mortality rats associated with chronic diseases in China, improve the health status of older adults, and enhance their overall quality of life. The logic model developed in this study serves as the theoretical foundation for the assessment system.

### Study setting

From November to December 2019, we conducted a demand-side survey by using self-designed questionnaires. A multi-stage stratified random sampling method was employed: (1) Province selection: Three provinces were selected, one from each region of China: Eastern (Zhejiang Province), Central (Shanxi Province), and Western (Chongqing Municipality). (2) City selection: Based on the economic development level, four prefecture-level cities and one municipality were selected. In Zhejiang Province and Shanxi Province, one city with a higher level of economic development and one with a lower level of economic development were selected. Chongqing City was directly included as a municipality. (3) CHC selection: In each city, one district or one county were randomly selected. Multiple CHCs and township health centers were then selected on the basis of a combination of local recommendations and random sampling. (4) Survey procedure: Local health commissions assisted in recruiting investigators for the on-site survey. At each survey site, patients with hypertension and diabetes who had recently received medical services were surveyed using paper-based questionnaires. Prior to the on-site survey, investigators received training to ensure the quality of the survey process (understanding and completeness of the survey content). Questionnaire quality control was implemented twice at both the township and county levels. If quality control failed, additional questionnaires were administered until the required sample size was reached. All questionnaires were uniquely coded and inserted into the database by the questionnaire company. All participants provided written informed consent prior to their inclusion in the study.

The following patients were included in the survey: (1) older adults aged >65 years with hypertension or diabetes, attending CHCs on the day of the survey and (2) patients who consented to participate in this survey. Older adults aged >65 years who had not received a diagnosis of hypertension and diabetes and those patients with severe mental illness were excluded from the survey.

### Reliability and validity test

The reliability and validity of the questionnaire were evaluated using Cronbach’s alpha coefficient and confirmatory factor analysis (CFA). Specifically, internal consistency was assessed by calculating Cronbach’s alpha coefficients with 95% confidence intervals for each model (30, 31). Cronbach’s alpha coefficient estimates should be at least ≥0.6 and ideally ≥0.8 (32).

CFA and structural equation modeling were employed to evaluate the construct, convergent, and discriminant validity of the questionnaire, both of which are widely recognized methods for evaluating the structural validity of a model. In this study, the maximum likelihood method was employed for parameter estimation in the assessment models for hypertension and diabetes. Construct validity was evaluated using multiple goodness-of-fit indices, which include three absolute fit indices and four comparative fit indices from the CFA. The following thresholds were considered acceptable for good construct validity: the chi-square to degrees of freedom (*X^2^/df*) ratio should be <3, the root mean square error of approximation (RMSEA) should be <0.05 (33, 34), the goodness-of-fit index (GFI) should be >0.9, the confirmatory fit index should be >0.9 (35), the normed fit index (NFI) should be >0.9, the non-normed fit index (NNFI) should be >0.9, and the incremental fit index should be >0.9 (36–38).

To assess the convergent validity of each factor, we evaluated the composite reliability (CR), average variance extracted (AVE), and factor loadings of each item. A widely accepted rule of thumb is that standard loading estimates should be ≥0.5. For CR, a value of >0.6 is considered acceptable, and a value of >0.7 is considered good (39). For AVE, a value between 0.36 and 0.5 is considered acceptable, and a value of >0.5 is considered good (40). Good discriminant validity was defined as the condition where the AVE for each construct exceeds the squared value of the correlation coefficient between pairs of variables (24).

### Statistical analysis

Statistical analyses were conducted using SPSS 26.0 and AMOS 26.0. Cronbach’s alpha coefficients, CR, and AVE values were calculated using SPSS, and CFA was conducted using AMOS. Descriptive data are presented as means ± standard deviations (x ± SD) and percentages (%).

## Results

### Participants

A total of 1,560 older patients with hypertension (46.2% men; mean age, 72.60 years) and 964 older patients with diabetes (40.4% men; mean age, 72.03 years) completed the survey. Table 1 presents the descriptive characteristics of the two patient samples.

**Table 1:** Frequency distribution of the sociodemographic characteristics of study participants.

Older patients with hypertension (*n*=1,560)			Older patients with diabetes (*n*=1,009)		
Demographic characteristic	No.	Percentage (%)	Demographic characteristic	No.	Percentage (%)
Sex			Sex		
Men	720	46.2	Men	389	40.4
Women	840	53.8	Women	575	59.6
Age (mean, range)	72.60	(65–100)	Age (mean, range)	72.03	(65–90)
Marital status			Marital status		
Married	1266	81.2	Married	761	78.9
Unmarried	27	1.7	Unmarried	16	1.7
Divorce	18	1.2	Divorce	10	1.0
Widowed	237	15.2	Widowed	168	17.6
Highest level of education			Highest level of education		
Primary school or less	988	63.3	Primary school or less	635	65.9
Junior high school	355	22.8	Junior high school	199	20.6
High school / junior college	114	7.3	High school / junior college	65	6.7
Bachelor’s degree	46	2.9	Bachelor’s degree	35	3.6
Master's degree and above	2	0.1	Master's degree and above	0	0.0
Income monthly per capita			Income monthly per capita		
Less than 2,000 yuan	823	52.8	Less than 2,000 yuan	464	48.1
2,000 ~ 5,000 yuan	547	35.1	2,000 ~ 5,000 yuan	382	39.6
5,001 ~ 10,000 yuan	121	7.8	5,001 ~ 10,000 yuan	80	8.3
More than 10,000 yuan	61	3.9	More than 10,000 yuan	30	3.1
Career			Career		
Practitioners of government organs and institutions	62	4.0	Practitioners of government organs and institutions	51	5.3
Professional and technical personnel	99	6.3	Professional and technical personnel	57	5.9
Administrative operations, administrative affairs, and other clerical staff	32	2.1	Administrative operations, administrative affairs, and other clerical staff	23	2.4
Commercial, service personnel	82	5.3	Commercial, service personnel	53	5.5
Agricultural, forestry, animal husbandry, fishery agricultural workers	910	58.3	Agricultural, forestry, animal husbandry, fishery agricultural workers	520	53.9
Production and transportation equipment operators	130	8.3	Production and transportation equipment operators	92	9.5
unemployed	209	13.4	unemployed	141	14.6
Others	28	1.8	Others	26	2.7
Medical insurance type			Medical insurance type		
Urban employees medical insurance	377	24.2	Urban employees medical insurance	251	26.0
Urban residents medical insurance	316	20.3	Urban residents medical insurance	230	23.9
Rural cooperative medical insurance	492	31.5	Rural cooperative medical insurance	280	29.0
Commercial insurance	1	0.1	Commercial insurance	1	0.1
Urban and rural residents medical insurance	376	24.1	Urban and rural residents medical insurance	222	23.0
Publicly funded medical care	3	0.2	Publicly funded medical care	6	0.6
No insurance	3	0.2	No insurance	4	0.4
Others	0	0.0	Others	4	0.4

No., Number of participants.

The majority of patients with hypertension and diabetes (81.2 and 78.9%, respectively) were married. More than half of the participants (63.3% of the patients with hypertensive and 65.9% of the patients with diabetes) had received primary education or less. Nearly half of the participants (52.8% of the patients with hypertension and 48.1% of the patients with diabetes) reported a monthly per capita income of <2000 RMB. The participants were ever involved in various occupations, including husbandry, fishery, and agriculture (58.3% of the patients with hypertension and 53.9% of the patients with diabetes) and production and transportation equipment work (8.3% of the patients with hypertension and 9.5% of the patients with diabetes), whereas 13.4% of the patients with hypertension and 14.6% of the patients with diabetes were unemployed. The four major types of medical insurance among the participants were basic medical insurance for urban employees (24.2% of the patients with hypertension and 26.0% of the participants with diabetes), basic medical insurance for urban residents (20.3% of the patients with hypertension and 23.9% of the patients with diabetes), new rural cooperative medical insurance (31.5% of the patients with hypertension and 29.0% of the patients with diabetes), and basic medical insurance for urban and rural residents (24.1% of the patients with hypertension and 23.0% of the patients with diabetes). Together, these four insurance schemes accounted for over 90% of the participants’ medical coverage (Table 1).

### Item selection and modification

To assess the interrelationships among the 40 selected items and determine whether further item reduction was necessary, two steps were taken to refine the items.

Step 1: Exclusion of items unsuitable for factor analysis. Three items were excluded from the analysis due to their inappropriate response categories for CFA, despite their relevance to the survey content. These items were as follows: “ways to view health records” (item 3, factor 1), “suggestions for free health checkups” (item 7, factor 2), and “reasons for not testing blood pressure/glucose in CHCs/THCs” (item 12, factor 4).

Step 2: To verify the factor structure of the questionnaire and evaluate the relationship between observed variables and their underlying latent constructs, CFA was performed on the remaining 37 items. The results indicated that some items required modification to more accurately and conveniently assess chronic disease management services for older patients. Items with Cronbach’s alpha coefficients of <0.5 and factor loadings of <0.5 were considered for deletion. Following expert group discussions on the content validity of these items, among the 37 items, the items “patient’s BMI” (item 13), “patient’s waist circumference” (item 14), “patient’s smoking status” (item 15), “patient’s alcohol consumption” (item 16), and “complications in patients” (item 17) were removed from the factor “examinations and assessment in follow-up” because their factor loadings were substantially lower than those of the others and were similar to baseline values rather than values obtained at follow-up assessments. The items “disease detection at CHCs/THCs” (item 6), “satisfaction with health record updates in follow-up visits” (item 35), and “satisfaction with the TCM treatment in follow-up visits” (item 39) were deleted due to their low validity scores, suggesting that they were more suitable for a more comprehensive study of factors influencing blood pressure and glucose follow-up. Finally, the response options for items 8, 9, 18, and 21 were modified to ensure that all the participants could provide suitable responses (Supplementary Table 1).

Through a two-step screening process, we developed a second-order factor assessment model. The final model comprises 29 items, which are categorized into three core areas of the NEPHSP: “essential health management services for all older adults,” “health management services for older adults with hypertension or diabetes,” and “self-assessment of patient experience satisfaction.” These categories encompass a total of nine factors: “health records and health education,” “annual examination,” “health management with TCM,” “blood pressure/glucose monitoring,” “examinations and assessment in follow-up,” “health coaching in follow-up,” “overall satisfaction with health management services for older patients,” “satisfaction with essential health services for all older adults,” and “satisfaction with follow-up services for older patients.” Table 2 presents the 29-item instrument used in the assessment model (Table 3).

**Table 2:** Definitions of domain and selected items in the assessment model.

Domains and factors	Item
**Service utilization by all older adults:** This refers to the utilization of essential services in the service package by all older adults, regardless of whether they have hypertension or diabetes. These services include health record establishment, health education programs, health checkups, and Chinese medicine health guidance.	
Health records and health education	Did the community/township health centers (CHCs/THCs) establish a health record for you?
	Have you ever participated in health education activities organized by the CHCs/THCs?
Annual physical examination	Have you participated in free health checkups organized by the CHCs/THCs once in the last year?
	Have you had ancillary examinations at a CHC/THC in the last year?
Health management through TCM	Have you received TCM identification for the constitution in the community within a year?
	Have you received TCM health-care guidance in the community within a year?
**Follow-up service utilization among older adults with hypertension or diabetes:** This refers to the utilization of follow-up services specifically designed for older adults with hypertension or diabetes. These services include the annual four follow-ups, which comprise including a blood pressure/blood glucose test, physical examination, symptom assessments, medication guidance, and lifestyle and integrated interventions.	
Blood pressure/glucose monitoring	Did you receive four follow-ups (including follow-ups at CHCs and through telephone and door-to-door visits) in the past year?
	Have you received a free blood pressure test from the CHCs/THCs during the follow-up in the past year?
Examinations and assessment during follow-up	Did the medical stuff examine your vital signs and enquire about your disease symptoms? (e.g., cardiac auscultation for hypertension, dorsalis pedis artery pulsation for diabetes)
	Did the medical staff inquire about the onset and development of symptoms related to comorbidities during the follow-up?
Health coaching during follow-up	Did the medical staff provide you with guidance on medication during the follow-up?
	Did the medical staff provide you with guidance on chronic disease knowledge during the follow-up?
**Assessment of patient satisfaction:** This involves evaluating patients’ satisfaction with their service experience and health outcomes facilitated by the health center and medical staff after receiving health management services. Unlike the first two domains, this domain focuses more on subjective perceptions of service quality rather than reporting the completion of specific health management processes.	
Overall satisfaction with health management services for older patients	Are you satisfied with the essential health services for all older adults?
	Are you satisfied with follow-up services?
	Are you satisfied with overall health management services in the NEPHSP?
Satisfaction with essential health services for all older adults	Are you satisfied with the service attitude of the medical examiners?
	Are you satisfied with the service level of medical examiners?
	Are you satisfied with the utilization of health records in CHCs/THCs?
	Are you satisfied with health education and promotion at CHCs/THCs?
	Are you satisfied with the TCM management?
	Are you satisfied with the content of the physical examination?
	Are you satisfied with the timely notification of physical examination results?
	Are you satisfied with the interpretation of the physical examination results?
Satisfaction with follow-up services for older patients	Are you satisfied with the service attitude of the medical staff in follow-up services?
	Are you satisfied with the service level of the medical staff in follow-up services?
	Are you satisfied with the physical examinations in follow-up services?
	Are you satisfied with the health coaching in follow-up services?
	Are you satisfied with the effect of blood pressure/glucose control in follow-up services?
	Are you satisfied with the screening for complications in follow-up services?

**Table 3:** Descriptive statistics of items in the two models.

Item	Response categories							
	Hypertension				Diabetes			
	Yes = 3 *N*(%)	Uncertain =2 *N*(%)	No = 1 *N*(%)	Missing	Yes = 3 *N*(%)	Uncertain =2 *N*(%)	No = 1 *N*(%)	Missing
Did the community/township health centers (CHCs/THCs) establish a health record for you?	1,394 (89.4)	125 (8.0)	35 (2.2)	6 (0.4)	858 (80.0)	72 (7.5)	30 (3.1)	4 (0.4)
Have you ever participated in health education activities organized by the CHCs/THCs?	1,303 (83.5)	142 (9.1)	84 (5.4)	31 (2.0)	807 (83.7)	61 (6.3)	96 (10.0)	0 (0.0)
Have you participated in free health checkups organized by the CHCs/THCs once in the last year?	1,457 (93.4)	16 (1.0)	87 (5.6)	0 (0.0)	902 (93.6)	1 (0.1)	61 (6.3)	0 (0.0)
Have you had ancillary examinations at a CHC/THC in the last year?	1,172 (75.1)	1 (0.1)	387 (24.8)	0 (0.0)	726 (75.3)	1 (0.1)	238 (24.6)	0 (0.0)
Have you received TCM identification for the constitution in the community within a year?	1,157 (74.2)	0 (0.0)	403 (25.8)	0 (0.0)	718 (74.5)	5 (0.5)	246 (25.0)	0 (0.0)
Have you received TCM health-care guidance in the community within a year?	879 (56.3)	0 (0.0)	681 (43.7)	0 (0.0)	508 (52.7)	5 (0.5)	458 (46.8)	0 (0.0)
Did you receive four follow-ups (including follow-ups at CHCs and through telephone and door-to-door visits) in the past year?	1,510 (96.8)	0 (0.0)	43 (2.8)	7 (0.4)	183 (18.8)	0 (0.0)	783 (81.2)	0 (0.0)
Have you received a free blood pressure test from the CHCs/THCs during the follow-up in the past year?	1,459 (93.5)	0 (0.0)	94 (6.0)	7 (0.4)	215 (22.3)	0 (0.0)	749 (77.7)	0 (0.0)
Did the medical stuff examine your vital signs and enquire about your disease symptoms? (e.g., cardiac auscultation for hypertension, dorsalis pedis artery pulsation for diabetes)	1,427 (91.5)	0 (0.0)	117 (7.5)	16 (1.0)	488 (50.6)	0 (0.0)	476 (49.4)	0 (0.0)
Did the medical staff inquire about the onset and development of symptoms related to comorbidities during the follow-up?	1,508 (96.7)	0 (0.0)	36 (2.3)	16 (1.0)	254 (26.3)	0 (0.0)	710 (73.7)	0 (0.0)
Did the medical staff provide you with guidance on medication during the follow-up?	1,396 (89.5)	0 (0.0)	164 (10.5)	0 (0.0)	275 (28.5)	0 (0.0)	689 (71.5)	0 (0.0)
Did the medical staff provide you with guidance on chronic disease knowledge during the follow-up?	1,523 (97.6)	0 (0.0)	37 (2.4)	0 (0.0)	238 (24.7)	0 (0.0)	726 (75.3)	0 (0.0)

	5	4	3	2	1	Missing	5	4	3	2	1	Missing
	Very satisfied–Very unsatisfied						Very satisfied–Very unsatisfied					
Are you satisfied with the essential health services for all older adults?	690 (44.2)	693 (44.4)	153 (9.8)	7 (0.4)	16 (1.0)	1 (0.1)	243 (25.2)	581 (60.3)	125 (13.0)	8 (0.8)	0 (0.0)	7 (0.7)
Are you satisfied with follow-up services?	520 (33.3)	880 (56.4)	119 (7.6)	28 (1.8)	3 (0.2)	10 (0.6)	223 (23.1)	627 (65.0)	102 (10.6)	9 (0.9)	1 (0.1)	2 (0.2)
Are you satisfied with overall health management services in the NEPHSP?	549 (35.2)	795 (51.0)	186 (11.9)	16 (1.0)	0 (0.0)	14 (0.9)	321 (33.3)	509 (52.8)	115 (11.9)	3 (0.3)	13 (1.3)	3 (0.3)
Are you satisfied with the service attitude of the medical examiners?	854 (54.7)	632 (40.5)	50 (3.2)	7 (0.4)	13 (0.8)	4 (0.3)	418 (43.4)	506 (52.5)	30 (3.1)	3 (0.3)	5 (0.5)	2 (0.2)
Are you satisfied with the service level of medical examiners?	714 (45.8)	755 (48.4)	74 (4.7)	6 (0.4)	9 (0.6)	2 (0.1)	345 (35.8)	555 (57.6)	58 (6.0)	2 (0.2)	2 (0.2)	2 (0.2)
Are you satisfied with the utilization of health records in CHCs/THCs?	512 (32.8)	707 (45.3)	251 (16.1)	6 (0.4)	81 (5.2)	3 (0.2)	239 (24.8)	468 (48.5)	207 (21.5)	4 (0.4)	40 (4.1)	6 (0.6)
Are you satisfied with health education and promotion at CHCs/THCs?	608 (39.0)	720 (46.2)	191 (12.2)	11 (0.7)	27 (1.7)	3 (0.2)	285 (29.6)	490 (50.8)	167 (17.3)	7 (0.7)	11 (1.1)	4 (0.4)
Are you satisfied with the TCM management?	755 (48.4)	657 (42.1)	111 (7.1)	8 (0.5)	26 (1.7)	3 (0.2)	338 (35.1)	497 (51.6)	115 (11.9)	1 (0.1)	10 (1.0)	3 (0.3)
Are you satisfied with the content of the physical examination?	726 (46.5)	662 (42.4)	132 (8.5)	8 (0.5)	25 (1.6)	7 (0.4)	333 (34.5)	472 (49.0)	143 (14.8)	2 (0.2)	11 (1.1)	3 (0.3)
Are you satisfied with the timely notification of physical examination results?	629 (40.3)	747 (47.9)	145 (9.3)	8 (0.5)	26 (1.7)	5 (0.3)	314 (32.6)	476 (49.4)	150 (15.6)	5 (0.5)	15 (1.6)	4 (0.4)
Are you satisfied with the interpretation of the physical examination results?	666 (42.7)	803 (51.5)	78 (5.0)	3 (0.2)	5 (0.3)	5 (0.3)	317 (32.9)	568 (58.9)	70 (7.3)	2 (0.2)	2 (0.2)	5 (0.5)
Are you satisfied with the service attitude of the medical staff in follow-up services?	975 (62.5)	539 (34.6)	38 (2.4)	3 (0.2)	4 (0.3)	1 (0.1)	443 (46.0)	490 (50.8)	29 (3.0)	1 (0.1)	0 (0.0)	1 (0.1)
Are you satisfied with the service level of the medical staff in follow-up services?	796 (51.0)	680 (43.6)	75 (4.8)	4 (0.3)	4 (0.3)	1 (0.1)	352 (36.5)	559 (58.0)	52 (5.4)	1 (0.1)	0 (0.0)	0 (0.0)
Are you satisfied with the physical examinations in follow-up services?	698 (44.7)	708 (45.4)	135 (8.7)	4 (0.3)	14 (0.9)	1 (0.1)	315 (32.7)	505 (52.4)	133 (13.8)	3 (0.3)	6 (0.6)	2 (0.2)
Are you satisfied with the health coaching in follow-up services?	886 (56.8)	604 (38.7)	57 (3.7)	1 (0.1)	11 (0.7)	1 (0.1)	408 (42.3)	489 (50.7)	63 (6.5)	0 (0.0)	0 (0.0)	4 (0.4)
Are you satisfied with the effect of blood pressure/glucose control in follow-up services?	763 (48.9)	648 (41.5)	107 (6.9)	31 (2.0)	8 (0.5)	3 (0.2)	285 (29.6)	478 (49.6)	139 (14.4)	58 (6.0)	2 (0.2)	2 (0.2)
Are you satisfied with the screening for complications in follow-up services?	793 (50.8)	701 (44.9)	60 (3.8)	3 (0.2)	2 (0.1)	1 (0.1)	346 (35.9)	566 (58.7)	49 (5.1)	2 (0.2)	0 (0.0)	1 (0.1)

### Internal consistency

The hypertension and diabetes models had satisfactory internal consistency. The Cronbach’s *α* coefficients for the three domains of the hypertension model were 0.613, 0.638, and 0.946, all exceeding the acceptable threshold of 0.6. The diabetes model had comparable results (Supplementary Table 2).

### Construct validity

A fundamental assumption of structural equation modeling and maximum likelihood estimation is the presence of a multivariate normal distribution. If this assumption is violated, the *X^2^* value derived from maximum likelihood estimation may be overestimated, and some fit indices may be modestly underestimated (37). Thus, the critical ratio of multivariate kurtosis was tested, with values of >5.00 indicative of nonnormal data distribution (41). In this study, the critical ratios for the hypertension and diabetes models were 480.101 and 177.004, respectively, indicating nonnormality in the data. To address this, the Bollen–Stine bootstrap method was applied to correct the *X^2^* value and goodness-of-fit indices of multivariate nonnormal data (42).

**Figure 2:**
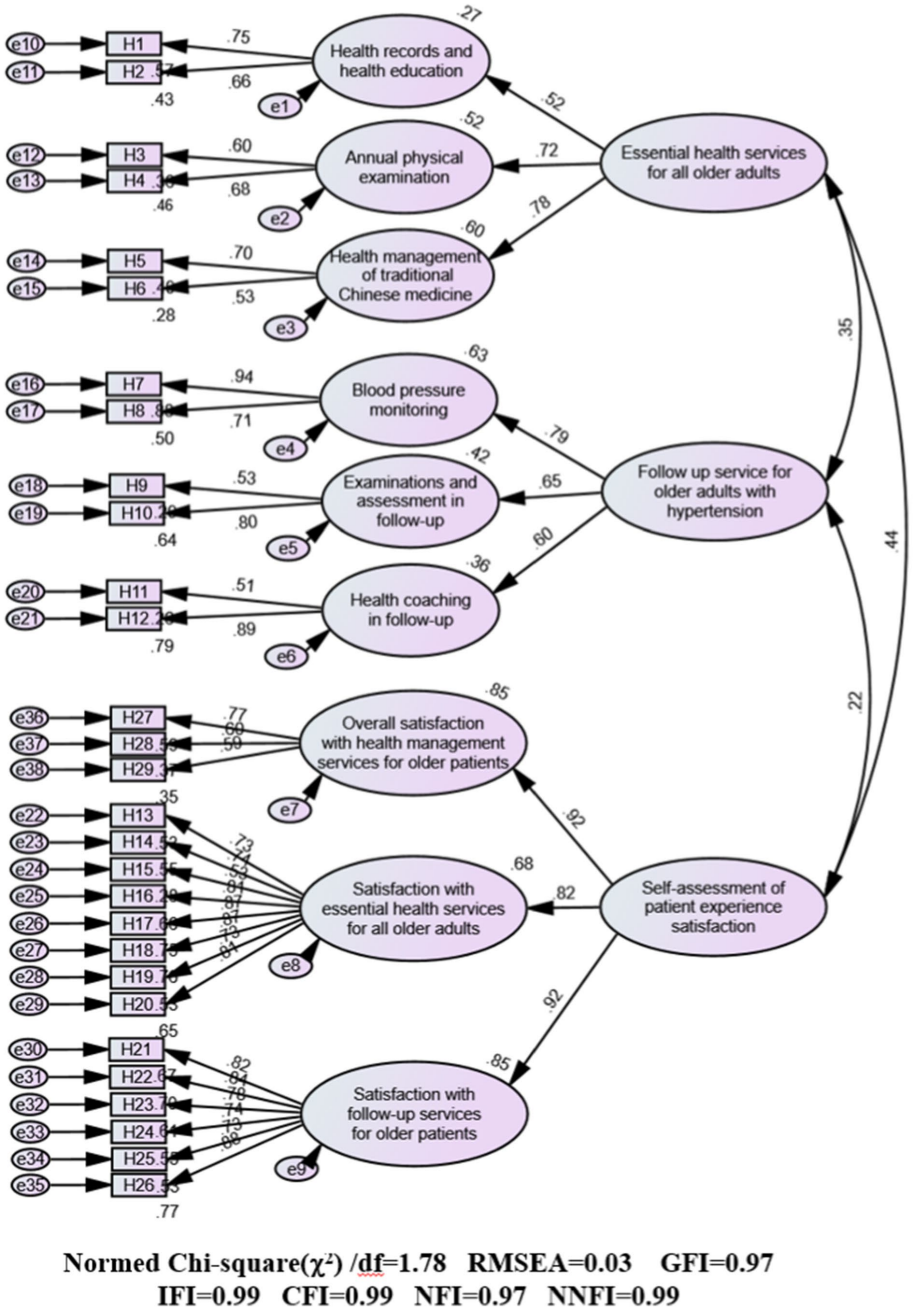
Standardized path diagram of confirmatory factor analysis (CFA) for the hypertension model.

**Figure 3:**
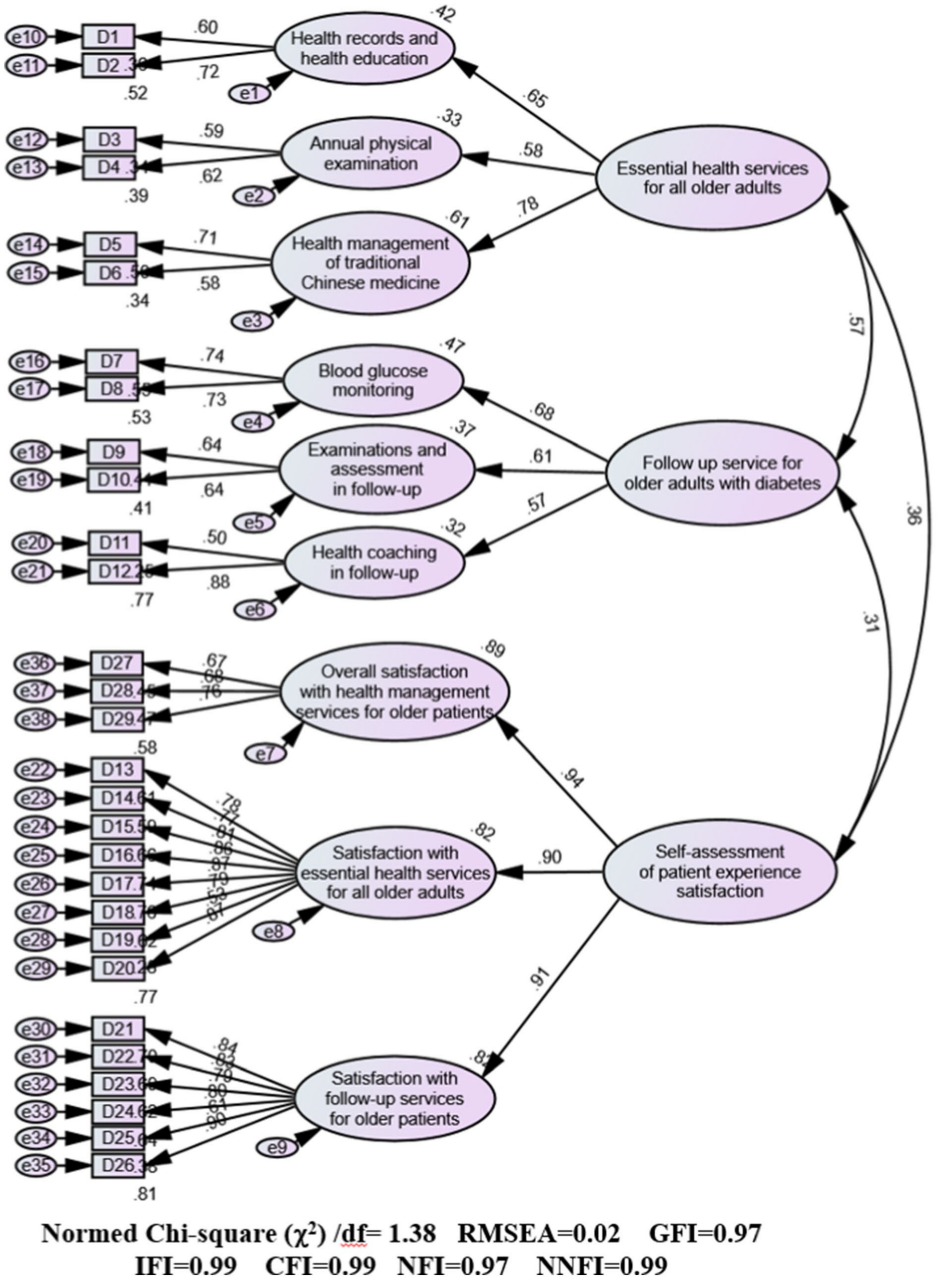
Standardized path diagram of confirmatory factor analysis (CFA) for the diabetes model.

The bootstrap analysis conducted for 2000 iterations revealed a relatively good fit between the proposed model and observed data (Figures 2, 3). For the hypertension model, the absolute fit indices were as follows: Bollen–Stine bootstrap *χ^2^/df* = 1.78, GFI = 0.97, and RMSEA = 0.03. The comparative fit indices were as follows: incremental fit index = 0.99, confirmatory fit index = 0.99, NFI = 0.97, and NNFI = 0.99. The *χ2/df* value of 1.38 was below the threshold of 3, the RMSEA value of 0.03 was below the accepted cutoff of 0.05, and all other indices exceeded the benchmark of 0.90, confirming a good model fit. Similarly, the diabetes model also demonstrated strong fit indices: Bollen–Stine bootstrap *χ2/df* = 1.38, GFI = 0.97, and RMSEA = 0.02. The comparative fit indices were incremental fit index = 0.99, confirmatory fit index = 0.99, NFI = 0.97, and NNFI = 0.99. The *χ^2^/df* value of 1.38 was lower than the threshold of 3 and the RMSEA value of 0.02 was below the threshold of 0.05, with all other indices exceeding 0.90. The fit indices across the three domains—“essential health management services for older adults,” “follow-up service for older adults with hypertension or diabetes,” and “self-assessment of patient experience satisfaction”—indicate that the resultant hypertension and diabetes models fit the data well.

### Convergent validity

The convergent validity results for the three-factor and item measures are detailed in Tables 4, 5. Factor loadings obtained from the CFA ranged from 0.516 to 0.940 for the hypertension model and from 0.504 to 0.943 for the diabetes model. These values indicate that each latent variable was well represented by its associated observed variables. The AVE ranged from 0.387 to 0.692 for the hypertension model and from 0.365 to 0.640 for the diabetes model, meeting the acceptable threshold of 0.36. Similarly, the CR values were acceptable, ranging from 0.553 to 0.920 for the hypertension model and from 0.535 to 0.941 for the diabetes model. The “annual physical examination” (AVE: 0.413 for the hypertension model and 0.365 for the diabetes model), “health management of TCM” (AVE: 0.387 for the hypertension model and 0.421 for the diabetes model), and “examinations and assessment in follow-up” (AVE: 0.460 for the hypertension model and 0.407 for the diabetes model) had borderline AVE values but were still within a reasonable fit range. Additionally, “overall satisfaction with health management services for older patients” (AVE: 0.435) in the hypertension model also had reasonable fit with borderline AVE. In summary, the three indicators—factor loadings, AVE, and CR—fall within acceptable ranges, confirming that the models have convergent validity.

**Table 4:** Item characteristics and factor loadings in the hypertension model.

Path	Estimate	S.E.	C.R.	*p*	CR	AVE
F4 ← F1	0.516				0.716	0.463
F5 ← F1	0.722	0.146	8.330	***		
F6 ← F1	0.776	0.161	8.488	***		
F7 ← F2	0.646	0.128	7.556	***	0.723	0.469
F8 ← F2	0.792	0.149	8.683	***		
F9 ← F2	0.602					
F10 ← F3	0.824				0.920	0.794
F11 ← F3	0.923	0.043	24.203	***		
F12 ← F3	0.923	0.045	19.463	***		
Item1 ← F4	0.755	0.083	10.524	***	0.666	0.500
Item2 ← F4	0.656					
Item3 ← F5	0.604	0.040	11.887	***	0.584	0.413
Item4 ← F5	0.679					
Item5 ← F6	0.701				0.553	0.387
Item6 ← F6	0.531	0.076	11.260	***		
Item7 ← F7	0.940				0.815	0.692
Item8 ← F7	0.708	0.054	20.268	***		
Item9 ← F8	0.534				0.621	0.460
Item10 ← F8	0.797	0.073	11.657	***		
Item11 ← F9	0.511				0.675	0.527
Item12 ← F9	0.890	0.080	10.802	***		
Item13 ← F10	0.731				0.918	0.589
Item14 ← F10	0.741	0.033	29.204	***		
Item15 ← F10	0.530	0.057	20.600	***		
Item16 ← F10	0.810	0.043	32.128	***		
Item17 ← F10	0.868	0.041	34.585	***		
Item18 ← F10	0.873	0.041	34.794	***		
Item19 ← F10	0.725	0.042	28.557	***		
Item20 ← F10	0.805	0.031	31.916	***		
Item21 ← F11	0.818				0.914	0.639
Item22 ← F11	0.836	0.028	38.975	***		
Item23 ← F11	0.781	0.035	35.357	***		
Item24 ← F11	0.744	0.032	33.068	***		
Item25 ← F11	0.728	0.036	32.091	***		
Item26 ← F11	0.879	0.026	42.028	***		
Item27 ← F12	0.770	0.068	22.215	***	0.695	0.435
Item28 ← F12	0.605					
Item29 ← F12	0.588	0.053	18.574	***		

S.E., standard error; C.R., critical ratio; CR, composite reliability; AVE, average variance extracted. * *p* < 0.05; ** *p* < 0.01; *** *p* < 0.001.

**Table 5:** Item characteristics and factor loadings in the diabetes model.

Path	Estimate	S.E.	C.R.	*p*	CR	AVE
F4 ← F1	0.646	0.545	6.514	***	0.710	0.454
F5 ← F1	0.578					
F6 ← F1	0.781	0.442	6.619	***		
F7 ← F2	0.609	0.128	7.076	***	0.653	0.387
F8 ← F2	0.684	0.119	7.748	***		
F9 ← F2	0.567					
F10 ← F3	0.904				0.941	0.842
F11 ← F3	0.906	0.041	22.456	***		
F12 ← F3	0.943	0.047	18.719	***		
Item1 ← F4	0.597	0.063	8.813	***	0.609	0.440
Item2 ← F4	0.724					
Item3 ← F5	0.585				0.535	0.365
Item4 ← F5	0.623	0.264	7.149	***		
Item5 ← F6	0.709				0.590	0.421
Item6 ← F6	0.582	0.097	9.667	***		
Item7 ← F7	0.744				0.702	0.540
Item8 ← F7	0.726	0.094	11.125	***		
Item9 ← F8	0.638				0.579	0.407
Item10 ← F8	0.638	0.110	8.005	***		
Item11 ← F9	0.878				0.662	0.513
Item12 ← F9	0.504	0.086	6.976	***		
Item13 ← F10	0.778				0.930	0.628
Item14 ← F10	0.771	0.036	26.040	***		
Item15 ← F10	0.527	0.066	16.725	***		
Item16 ← F10	0.810	0.048	27.707	***		
Item17 ← F10	0.863	0.044	30.072	***		
Item18 ← F10	0.870	0.046	30.406	***		
Item19 ← F10	0.788	0.050	26.776	***		
Item20 ← F10	0.875	0.035	30.633	***		
Item21 ← F11	0.837				0.913	0.640
Item22 ← F11	0.833	0.032	31.842	***		
Item23 ← F11	0.786	0.043	29.094	***		
Item24 ← F11	0.802	0.038	29.993	***		
Item25 ← F11	0.614	0.054	20.744	***		
Item26 ← F11	0.899	0.031	36.100	***		
Item27 ← F12	0.761	0.072	20.050	***	0.748	0.499
Item28 ← F12	0.683	0.053	18.355	***		
Item29 ← F12	0.671					

S.E., standard error; C.R., critical ratio; CR, composite reliability; AVE, average variance extracted. * *p* < 0.05; ** *p* < 0.01; *** *p* < 0.001.

### Discriminant validity

The models had discriminant validity. The analysis revealed that all the three first-order factors were significantly correlated with each other (*p* < 0.01), with their correlation coefficients lower than the square root of AVE for each respective factor in both models (Table 6). This finding indicates that although the latent variables exhibit some degree of correlation, they remain sufficiently distinct from one another.

**Table 6:** Bivariate correlations of three constructs in the two models.

Hypertension model				Diabetes model			
	F1	F2	F3		F1	F2	F3
F1	1			F1	1		
F2	0.351***	1		F2	0.568***	1	
F3	0.435***	0.221***	1	F3	0.311***	0.358***	1
The square root of AVE	0.681	0.685	0.891	The square root of AVE	0.674	0.622	0.918

*** *p* < 0.001.

## Discussion

This study developed and validated the questionnaire designed to assess the utilization of the NEPHS from the perspective of older patients. The questionnaire is a comprehensive tool focused on patients’ accessibility to and satisfaction with health management services delivered through community-based interventions rather than traditional organizational measures or monitoring frameworks (43–45). The CFA, internal consistency assessment, and intercorrelation analyses across the 29 items demonstrated that the instrument is valid and reliable. These findings were consistent across various cities in regions with different levels of socioeconomic development in China.

This questionnaire is considered suitable for use not only in China but also in other countries. Health administration authorities can employ this tool to assess the effectiveness of public health programs in a phased manner, enhancing and supporting chronic disease management among older patients and ultimately improving their quality of life. For health-care providers, the 29-item scale offers three notable advantages. First, the study adopted a demand-side perspective, and the scale may more efficiently capture the items or domains that may be challenging to assess through service providers, particularly direct patient benefit variables rather than indirect process indicators. For example, evaluating the effectiveness of health education, literacy, and guidance is difficult because the outcome can substantially differ between project monitoring metrics and patients’ subjective experiences (46). Second, this assessment system encompassed the core health management services provided to older adults with chronic conditions under the NEPHSP. This comprehensive coverage facilitates comparisons of service effectiveness across different health-care centers over time. Third, considering the digital divide among older populations and their preference for on-site, paper-based surveys over online formats (47, 48), the questionnaire is designed with less technical language and has a hard copy version. This format is particularly suited to older adults in the study sample, most of whom did not have a high education level. Additionally, investigators are trained to implement relevant measures to assist participants in completing the questionnaire. Built on this foundation, the assessment tool is expected to be comprehensible, intuitive, and relevant to patient experiences.

### Limitations

Despite the recruitment of a large and diverse sample, the study was limited to patients attending community health-care centers rather than encompassing a broader section of the population. Consequently, survey responses may reflect more favorable assessments of health examination and monitoring services among older patients, whose health management patterns may not fully represent those of broader or clinically managed populations. Consequently, this validated tool will continue to be used for assessing the effectiveness of chronic disease management and long-term monitoring programs, with plans for nationwide implementation in China. In developing the item pool, the focus was on capturing patients’ actual utilization of core services within the NEPHSP and their satisfaction with service processes, outcomes, and provider attitudes. However, items addressing ethical implications and professional responsibilities during service delivery, which are crucial to the patient experience, were not included. Continuous NEPHSP services for older patients with chronic disease depend on dynamically updated electronic health records containing sensitive personal information, such as annual physical examination data and quarterly follow-up results (49). This raises concerns regarding the core ethical and professional responsibilities of medical professionals, particularly regarding the safeguarding of such sensitive data (50). Future studies will address this gap by incorporating patients’ perspectives on these critical concerns into the assessment tool, refining its scope to better capture this essential aspect of health care.

## Conclusion

In this study, a valid and reliable tool for assessing health management service utilization among older adults with hypertension and diabetes was developed. Data from a large and diverse sample of patients revealed very good to excellent fit by the logical model. The tool serves as a foundational framework for further tool development and holds promise for broader application in the evaluation of essential public health and primary health-care services. However, further validation in diverse health-care settings is required to fully establish its generalizability and effectiveness.

## Data Availability

The original contributions presented in the study are included in the article/Supplementary material, further inquiries can be directed to the corresponding author.
