# Supplementary material for: A patient-reported questionnaire for evaluating utilization of the National Essential Public Health Service Program in China among older adults with hypertension and diabetes
Source: Front Public Health. 2025 Mar 11;13:1459754. doi: 10.3389/fpubh.2025.1459754 (PMC11932853; doi:10.3389/fpubh.2025.1459754)
Supplement: Supplementary file 1 [file Table_1.DOCX]

**Supplementary materials**

**Supplementary Table 1. Domains and original 40 potential items for assessing services utilization among older adults with hypertension or diabetes within the NEPHSP**

| **Domain and factor** |  | **Item** |
| --- | --- | --- |
| **Service utilization by all older adults:** This refers to the utilization of essential services in the service package by all older adults, regardless of whether they have hypertension or diabetes. These services include health record establishment, health education programs, health checkups, and Chinese medicine health guidance. | | |
| Health records and health education | Item1 | Did the community/township health centers (CHCs/THCs) establish a health record for you? |
|  | Item2 | Have you ever participated in health education activities organized by the CHCs/THCs? |
|  | Item3 | Which of the following channels have you used to view your health records, paper records, cell phone (App/We Chat), computer (e-mail/electronic files) or doctor's workstation? |
| Annual physical examination | Item4 | Have you participated in free health checkups organized by the CHCs/THCs once in the last year? |
|  | Item5 | Have you had ancillary examinations at a CHC/THC in the last year? |
|  | Item6 | Have you been screened for diseases through health checkups in the CHCs/THCs? |
|  | Item7 | Do you recommend increasing or decreasing the number and frequency of health checkups? |
| Health management of traditional Chinese medicine (TCM) | Item8 | Have you received TCM identification for the constitution in the community within a year? |
|  | Item9 | Have you received TCM health care guidance in the community within a year? |
| **Follow-up service utilization among older adults with hypertension or diabetes:** This refers to the utilization of follow-up services specifically designed for older adults with hypertension or diabetes. These services include the annual four follow-ups, which comprise including a blood pressure/blood glucose test, physical examination, symptom assessments, medication guidance, and lifestyle and integrated interventions. | | |
| Blood pressure/glucose monitoring | Item10 | Did you receive four follow-ups (including follow-ups at CHCs and through telephone and door-to-door visits) in the past year? |
|  | Item11 | Have you received a free blood pressure test from the CHCs/THCs during the follow-up in the past year? |
|  | Item12 | The reasons you did not have free blood pressure/glucose monitoring at the CHCs/THCs include which of the following? |
| Examinations and assessment during follow-up | Item13 | What is your BMI? |
|  | Item14 | What is your waist circumference? |
|  | Item15 | How often do you smoke? |
|  | Item16 | What is your daily alcohol consumption? |
|  | Item17 | Do you already suffer from complications? |
|  | Item18 | Did the medical stuff examine your vital signs and enquire about your disease symptoms? (e.g., cardiac auscultation for hypertension, dorsalis pedis artery pulsation for diabetes) |
|  | Item19 | Did the medical staff inquire about the onset and development of symptoms related to comorbidities during the follow-up? |
| Health coaching during follow-up | Item20 | Did the medical staff provide you with guidance on medication during the follow-up? |
|  | Item21 | Did the medical staff provide you with guidance on chronic disease knowledge during the follow-up? |
| **Assessment of patient satisfaction:** This involves evaluating patients’ satisfaction with their service experience and health outcomes facilitated by the health center and medical staff after receiving health management services. Unlike the first two domains, this domain focuses more on subjective perceptions of service quality rather than reporting the completion of specific health management processes. | | |
| Overall satisfaction with health management services for older patients | Item22 | Are you satisfied with the essential health services for all older adults? |
|  | Item23 | Are you satisfied with follow-up services? |
|  | Item24 | Are you satisfied with overall health management services in the NEPHSP? |
| Satisfaction with essential health services for all older adults | Item25 | Are you satisfied with the service attitude of the medical examiners? |
|  | Item26 | Are you satisfied with the service level of medical examiners? |
|  | Item27 | Are you satisfied with the utilization of health records in CHCs/THCs? |
|  | Item28 | Are you satisfied with health education and promotion at CHCs/THCs? |
|  | Item29 | Are you satisfied with the TCM management? |
|  | Item30 | Are you satisfied with the content of the physical examination? |
|  | Item31 | Are you satisfied with the timely notification of physical examination results? |
|  | Item32 | Are you satisfied with the interpretation of the physical examination results? |
| Satisfaction with follow-up services for older patients | Item33 | Are you satisfied with the service attitude of the medical staff in follow-up services? |
|  | Item34 | Are you satisfied with the service level of the medical staff in follow-up services? |
|  | Item35 | Are you satisfied with the updating of your health record in follow-up services? |
|  | Item36 | Are you satisfied with the physical examinations in follow-up services? |
|  | Item37 | Are you satisfied with the health coaching in follow-up services? |
|  | Item38 | Are you satisfied with the effect of blood pressure/glucose control in follow-up services? |
|  | Item39 | Are you satisfied with the TCM treatment in follow-up services? |
|  | Item40 | Are you satisfied with the screening for complications in follow-up services? |

| **Supplementary Table 2. The Cronbach’s α coefficients of two models** | | |
| --- | --- | --- |
| **Domains** | **Cronbach’s α coefficients** | |
|  | **Diabetes** | **Hypertension** |
| **Essential health services for all older adults** | 0.613 | 0.626 |
| **Follow-up service for older adults with hypertension or diabetes** | 0.638 | 0.654 |
| **Self-assessment of patient experience satisfaction** | 0.946 | 0.939 |
